# Interaction of Temporal Lobe Epilepsy and Posttraumatic Stress Disorder: Network Analysis of a Single Case

**DOI:** 10.3389/fpsyg.2020.01010

**Published:** 2020-05-20

**Authors:** Iftah Biran, Roee Admon, Tomer Gazit, Firas Fahoum

**Affiliations:** ^1^Neurological Institute, Tel Aviv Sourasky Medical Center, Tel Aviv, Israel; ^2^The Israel Neuropsychoanalysis Society, Kadima, Israel; ^3^Department of Psychology, University of Haifa, Haifa, Israel; ^4^Sagol Brain Institute, Tel Aviv Sourasky Medical Center, Tel Aviv, Israel; ^5^Sackler School of Medicine, Tel Aviv University, Tel Aviv, Israel

**Keywords:** PTSD, TLE, PNES, conversion disorder, fear circuitry

## Abstract

In this case study, we present a 21 years old female with long-standing Temporal Lobe Epilepsy (TLE) who, following a sexual assault, also developed Posttraumatic Stress Disorder (PTSD), leading to a change in her seizure semiology. The new seizures seemed to be a re-enactment of the sexual assault and accordingly were at first thought to be Psychogenic Non-Epileptic Seizures (PNES). Nevertheless, electroencephalography (EEG) recording at the Epilepsy Monitoring Unit (EMU) revealed ictal epileptic brain activity during these new attacks. In order to further explore the nature of the relation between epileptic seizures and PTSD symptomatology, a functional MRI (fMRI) scan was conducted focusing on neural response to threat (fearful faces). The results indicated that the response to threat elicited bilateral amygdala activation, as well as enhanced amygdala connectivity with the insula and anterior cingulate cortex (ACC), all central nodes of the fear circuitry. Accordingly, we suggest that this unique presentation of “pseudo” PNES might stem from the anatomical proximity of the epileptic network in this patient (temporal-insular-frontal) to the fear circuitry, allowing abnormal epileptic activity to “exploit” or activate the fear circuit or vice versa. We further propose that the traumatic experience may have changed the patient’s ictal semiology by modifying the course of the spread of the ictal activity toward the PTSD network.

## Introduction

Temporal Lobe Epilepsy (TLE) and posttraumatic stress disorder (PTSD) have been rarely reported to co-occur. A PubMed search performed on August 11, 2018 with PTSD or Post Traumatic Stress Disorder and TLE or Temporal Lobe Epilepsy or complex partial seizures as keywords yielded only eight relevant hits, including five case reports (Stewart and Bartucci, 1986; Smith et al., 2008; Cohen et al., 2010; Koubeissi, 2012; Cuomo et al., 2017), two case series with a total of 25 cases (Greig and Betts, 1992; de Barros et al., 2018), and a theoretical paper (Moreno and Peel, 2004). These publications discuss various elements of the association between TLE and PTSD; including a traumatic event leading to the activation and onset of an epileptic disorder (Greig and Betts, 1992), TLE being misdiagnosed as PTSD (Stewart and Bartucci, 1986; Koubeissi, 2012), and PTSD secondary to TLE ictal experiences (Cohen et al., 2010). In addition, more general aspects are also mentioned, including the epidemiology of TLE-PTSD co-occurrence (de Barros et al., 2018), diagnostic considerations between TLE and PTSD in the evaluation of patients following head trauma (Moreno and Peel, 2004), and clinical pharmacological interventions suggested for this co-occurrence (Cuomo et al., 2017).

TLE being misdiagnosed as PTSD and vice versa stem from several common characteristics shared by these two conditions, that may, at times, lead to confusion (Stewart and Bartucci, 1986; Greig and Betts, 1992; Moreno and Peel, 2004; Cohen et al., 2010; Koubeissi, 2012). These similarities are evident in the clinical presentation, as well as in the related neuroanatomy and the suggested brain mechanisms involved. Clinically – In both disorders memories can be stripped of their temporal context and therefore be misattributed in time: PTSD patients re-live traumatic memories as though they are experienced in the present (flashbacks, memory intrusions) (Brewin et al., 2010), while patients with TLE may attribute or misattribute current events to the past (e.g., Déjà vu experiences) (Warren-Gash and Zeman, 2014). Some authors even suggest that TLE-like clinical symptoms appear in an attenuated form in healthy populations, whereas in clinical psychiatric populations including PTSD, these symptoms are more prevalent and prominent (Persinger and Makarec, 1993). Concerning their neural representations, both disorders involve abnormalities in anterior mesial temporal lobe structures and brain networks that include the temporal lobes (Fiddick, 2011; Fahoum et al., 2012; Cisler et al., 2014; Maneshi et al., 2014; Boccia et al., 2016). More specifically, heightened amygdala (located in the temporal lobe) activation in response to aversive stimuli is considered central to PTSD pathophysiology, alongside aberrant anterior cingulate cortex (ACC) and insula activation (Admon et al., 2013b; Shalev et al., 2017).

In this paper we present a case-study of a patient with longstanding TLE who, following a traumatic event, presented with a new epileptic semiology mimicking a dissociative-flashback PTSD-like clinical phenomenon. This new presentation was initially thought to be Psychogenic Non-Epileptic Seizure (PNES), yet electroencephalography (EEG) evaluation at the Epilepsy Monitoring Unit (EMU) revealed ictal epileptic brain activity during these new attacks. This peculiar presentation, therefore, represents a unique and rare opportunity to investigate the neural interactions between the two similar yet very distinct conditions of TLE and PTSD. In order to address this, the patient underwent a functional MRI (fMRI) scan during which activation and connectivity of the fear circuitry were probed. The study was approved by the Tel Aviv Sourasky Medical Center (TASMC) Ethics committee, and the participant gave her written and informed consent for the publication of this case report and the use of her images.

## Case Report

### Past and Current History

A 21 years old right-handed woman, from Christian Arab ancestry, who was born in a cesarean section following an uneventful pregnancy. Her early development was normal, and she achieved the developmental milestones in time. One second-degree cousin had epilepsy. There was no history of head trauma. She graduated from high school, and, during her current assessment, was a college student.

Febrile seizures started at the age of 11 months and were treated with valproate until the age of 5 years. Between the ages of 5–16 years, the patient was seizure-free. Focal seizures started at the age of sixteen, characterized by altered consciousness with oral and bimanual automatism lasting up to a minute. A non-specific frontal cephalic aura preceded the seizures. The patient started Lamotrigine treatment with an increase of dose up to 400 mg/day, with no documented seizures at all. This typical seizure semiology was constant until the age of twenty.

At the age of twenty, approximately 6 months following a sexual assault by an unfamiliar man, the patient began suffering from nocturnal seizures with hyper-motor semiology accompanied by screaming, at the frequency of one to two events per week. She presented with right-hand automatism (fondling her genitalia) as well as verbal automatism (saying “take him away from me” and “no, dad”). At times she would hide under her blanket during seizures. She had no recollection of these events afterward (see Figure 2). There were no subjective memory problems, yet she reported anxiety symptoms and referred to psychotherapy.

### Neurological Evaluation and Follow-Up

**FIGURE 1:**
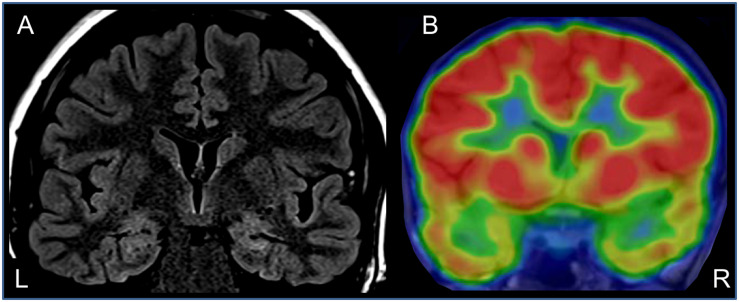
Anatomical and functional neuroimaging – coronal sections through the temporal lobes. **(A)** T2-weighted FLAIR MRI showing right hippocampal hyperintensity. **(B)** Interictal brain PET-CT – showing bilateral hypo-metabolism in temporal lobes (right >> left).

**FIGURE 2:**
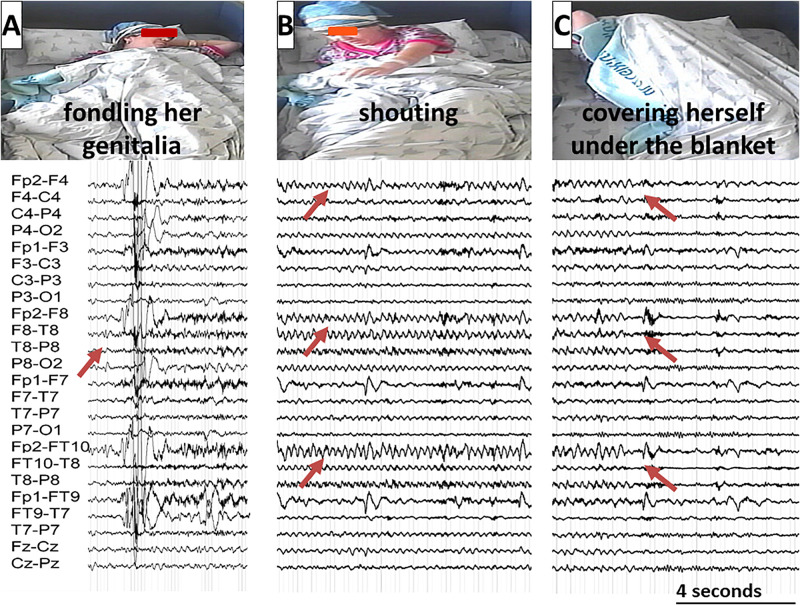
A typical seizure sequence lasting approximately 3 min with the corresponding EEG recording illustrated from left to right: **(A)** The patient is fondling her groin. The Arrow marks the ictal EEG onset over the right temporal regions; **(B)** The patient is shouting. The Arrows mark the seizure spread over the right frontotemporal regions; **(C)** The patient is covering herself under her blanket. The arrows mark the seizure end. (The patient gave her written and informed consent for the use of these images in the publication).

Neurological examination was unremarkable. Overall intellectual functioning was at the average range (25th percentile) both for visuospatial tasks and for verbal tasks. Performance on word list recall and memory for faces was within the normal range for her age. Standard EEG recording demonstrated inter-ictal epileptiform discharges and mild intermittent slowing over the right temporal region. T2-weighted fluid-attenuated inversion recovery (FLAIR) MRI scan revealed hyperintense signal in the mesial aspect of the right temporal lobe (Figure 1A), and PET-CT demonstrated bilateral temporal hypo-metabolism, more prominent on the right side (Figure 1B). Carbamazepine with a dose of up to 500 mg/d was added to her usual Lamotrigine treatment, and she was referred to the EMU for evaluation of the new type of events (Hyper-motor seizures vs. Psychogenic non-epileptic seizures). At EMU, after the gradual withdrawal of Carbamazepine, there were two ictal events; both were with vocalization, automatism, and resembled the newly described semiology. Ictal EEG showed right temporal epileptic activity spreading quickly over the right frontotemporal leads (Table 1 and Figure 2). Interictal EEG was significant for right temporal epileptiform discharges and mild intermittent slowing. The dual therapy of Lamotrigine and Carbamazepine was not effective in controlling seizures, with up to 2–3 focal seizures with impaired consciousness per month, and the treatment scheme was gradually switched to Lacosamide 500 mg/d and Clobazam 30 mg/d. During 3 years of follow-up in the epilepsy outpatient clinic, the patient and her family members reported the occurrence of up to 2–3 occasional seizures per year, all with the “new” semiology, and no seizures with the “old” semiology.

**TABLE 1:** Clinical details of the ictal events recorded at the Epilepsy Monitoring Unit (EMU).

Seizure #	Time from ictal EEG onset	Behavioral change	EEG change
1	00:00	–	F8, T8 sharp wave
	00:02	Wakes up, covering her face with the bed cover, right Manual and bi-pedal automatism	Movement artifact and F8, T8, F4, C4 semi-rhythmic slow waves
	00:07	–	F8, T8 rhythmic 2 Hz activity
	00:16	Bi-pedal automatism, pedaling, left manual automatism	–
	00:33	–	F8, T8, F4, C4 rhythmic 6 Hz activity
	00:34	Ictal scream, fear, shouting “No father,” hyper-motor behavior	–
	00:51	–	F8, T8, P8 rhythmic 4 Hz activity with sharp waves
	01:23	Oral automatism	–
	02:23	–	F8, T8 Intermittent slowing
	03:39	Oral automatism stops	–
2	00:00	Lying in bed, opens eyes	–
	00:01	–	F8, T8, P8 rhythmic theta
	00:04	Oral automatism, right hand automatism (fondling her groin)	–
	00:09	–	F8, T8, P8, F4, Fp2 rhythmic delta and spikes
	00:13	Calling her mother, calling her sister by name	–
	00:21	Moving in bed, looking frightened, screaming “Why”	–
	00:53	Moving in bed, oral automatism, partially following commands	–
	01:39	Not responding to nurse	–

### Psychiatric Evaluation

Psychiatric workup established the diagnosis of PTSD, based upon clinical interview and structured evaluation (Weathers et al., 2013a). The patient met the following DSM-5 criteria for PTSD: A – Exposure to a traumatic event (A1); B – Intrusion symptoms (B1,3-5); C – Avoidance symptoms (C1-2); D – Cognition and mood change (D1-4); E – Arousal and reactivity (E3-5); The disorder lasted more than a month (F criteria), caused significant distress (G criteria) and was not attributed to other etiology (H criteria) (American Psychiatric Association, 2013). Her PTSD Checklist for DSM-5 (PCL-5) score was 12, suggestive of mild PTSD symptomatology (Weathers et al., 2013b). Aside from the PTSD symptomatology, the patient had minimal anxiety and no apparent depression, with scores of 6 and 3 on the Beck Anxiety Inventory and Beck Depression Inventory-II respectively (Beck et al., 1988, 1996). She was prescribed with a Selective Serotonin Reuptake Inhibitor, which she declined to take and was referred to psychotherapy.

## Assessment of PTSD Fear Circuitry Responsivity

The pathophysiology of PTSD involves disruptions in various neural circuitries, including the ones that mediate threat detection, emotional regulation, and context processing (Sheynin and Liberzon, 2017). Among these circuitries, hyper responsivity to threat is considered a key component of PTSD to the extent that some authors claim that PTSD could be regarded as a stress-induced fear circuitry disorder (Bracha, 2006; Kim et al., 2011). Threat elicits substantial neural responses, with activation in the amygdala, insula, and ACC being most prominent (i.e., fear circuitry). In PTSD, hyper-responsivity to threat is believed to involve hyper amygdala activation as well as dysfunctional amygdala connectivity with other structures of the fear circuitry, putatively leading to inefficient regulation of fear (Etkin and Wager, 2007; Liberzon and Sripada, 2007).

### Methods

In order to assess the responsivity of fear circuitry in the patient, she underwent a functional MRI scan while engaged in a well-validated fMRI task that involves the presentation of fearful and neutral facial expressions. This task was previously shown to elicit amygdala activation (Hariri et al., 2002; Cohen et al., 2013). The scan was conducted on a 3T Siemens PRISMA MRI scanner with a 20-channel head coil. 195 functional volumes were acquired [TR = 2 s; 36 slices]. fMRI data were analyzed using SPM12 and included standard pre-processing followed by whole-brain analyses of activation and Psycho-Physiological Interaction (PPI) analyses of functional connectivity patterns in response to negative facial expression.

### Results

**FIGURE 3:**
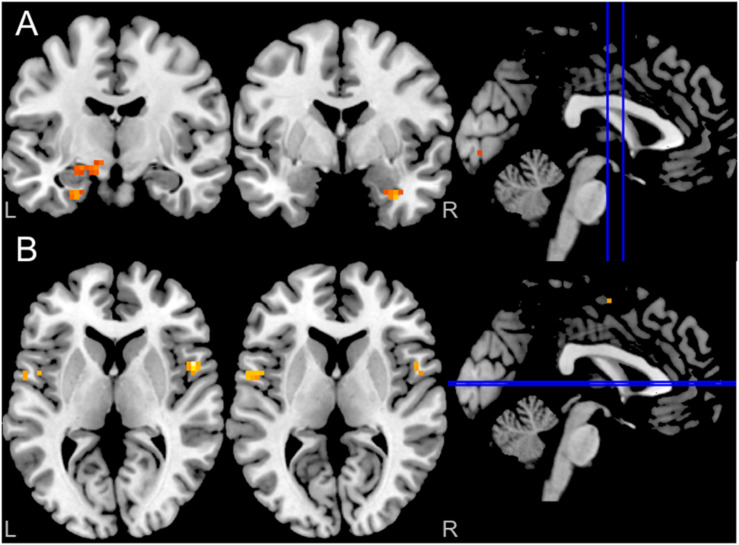
**(A)** Blood oxygenation level-dependent (BOLD) in response to fearful facial expressions demonstrated increased bilateral amygdala activity (*P* < 0.001 uncorrected for ten voxels); **(B)** Enhanced left amygdala connectivity (PPI) with both insula in response to fearful facial expressions (*P* < 0.001 uncorrected for ten voxels). The right amygdala did not show any PPI connectivity (not shown in the figure).

Activation and connectivity effects were thresholded at peak *P* < 0.001 uncorrected for ten voxels. Results indicate, as expected, bilateral amygdala activation in response to fearful compared to neutral faces (Figure 3A). Interestingly, effective connectivity analyses using PPI further revealed enhanced connectivity of the left (but not the right) amygdala with central nodes of the fear circuitry, including bilateral insula and ACC, in response to fearful compared to neutral faces (Figure 3B).

## Discussion

We present a clinical and neural examination of a patient whose rare presentation of epileptic seizures seemed to be related to PTSD and was correlated with abnormal epileptic activity. Conceptually, the interaction between epileptic seizures and PTSD for this patient can be bidirectional: either that epileptic seizures activate the fear network, or that PTSD symptoms that are associated with dysfunction in the fear network activate the TLE network and lead to a seizure. We suggest that this interaction might be related to one or a combination of the following interacting factors:

**The first** – The anatomical proximity (and potential overlap) of the fear network (Cisler et al., 2014) and the TLE network (Fahoum et al., 2012; Maneshi et al., 2014), both involving temporal-insular-frontal brain regions, enabled in the current case the TLE activity to exploit the newly kindled fear network (or vice versa). In support of that, cortical proximity and neuronal crosstalk as a causative explanation to symptom generation and transformation can be found in various conditions, both acquired and genetic. For instance, following hand amputation, the perceived location of the phantom limb can often be traced to the face area due to the anatomical proximity between the hand and the cortical face representations (Ramachandran and Blakeslee, 1998; Ramachandran and Hirstein, 1998). One of the postulated mechanisms for synesthesia, a developmental condition where sensory stimuli from one modality activate a perceptual system from a different modality, is the activation of adjacent cortical areas (Bargary and Mitchell, 2008).

**The second** – The interaction between these two adjacent networks could potentially be mediated via abnormal kindling of limbic networks through PTSD activity. This proposed mechanism is supported by animal studies that show that amygdala excitation reinforces learned fear behaviors (Kellett and Kokkinidis, 2004) as well as by evidence that epileptic network changes can lead to alteration in seizure semiology (Bartolomei et al., 2017). Following this line of thinking, such abnormal kindling can also modulate the epileptic network and even completely replace the “old” semiology with a “new” one. In this regard, PTSD induced alteration of limbic-frontal connectivity might facilitate seizure spread from mesial temporal to frontal and anterior insular brain regions. Such expansion of the epileptic network, in turn, may account for the new semiology considering that hyperkinetic behavior, shouting/screaming vocalization, and sexual automatisms are more often seen in frontal seizures and can mimic PNES (Kanner et al., 1990).

**The third** – Regardless of the anatomical proximity, this interaction could also be related to the psychological reaction to stress. It could be argued that the epileptic activity might have an affective negative bias (Clark, 1920; Gunn and Baram, 2017; Lang et al., 2018), and that this bias can lead to increased stress and anxiety thus triggering PTSD related anxiety (Cohen et al., 2010) forming a vicious cycle. In this regard, PTSD related stress might lead to a reactive seizure and vice versa (Greig and Betts, 1992).

This case could be formulated as stress-induced epilepsy, which can mimic PNES in part because of seizure semiology, in part because of interictal anxious traits (McGonigal and Bartolomei, 2014). However, it could also be regarded, albeit with much reservation, as a case of conversion disorder with atypical PNES. The co-occurrence of epileptic brain activity with the new ictus as our patient presented with is indeed not compatible with current definitions of PNES (Brown and Reuber, 2016), and as such, this case could not be formulated neither diagnosed as PNES. However, by using a broader and more classic definition of conversion disorder, our case is somewhat compatible with such diagnosis. The classic literature on conversion disorders suggests that the essence of these disorders is when a non-emotional neurological system or function sub-serves an emotional conflictual or aberrant system (Freud, 1910; Breuer and Freud, 1893-1895; American Psychiatric Association, 1952). Furthermore, according to current diagnostic formulations, the presence of a neurological diagnosis does not exclude the possibility of a conversion disorder (American Psychiatric Association, 2013). In this regard, in our case, the traumatic reminiscences “hijacked” not a healthy neurological system but rather the unhealthy TLE circuitry. The “hijacking” was facilitated through the anatomical proximity of the PTSD and the TLE circuitries. This can serve as a model for understanding the known co-occurrence of PNES with epilepsy with a prevalence of 9–50% of documented epilepsy in patients with PNES (Benbadis et al., 2001). This anatomic proximity explanation differs from previous observations of distinct PNES generators (Arzy et al., 2014).

This case study contains some limitations that need to be considered in the interpretation of these results and observations. First, as we had only limited collateral information regarding the actual sexual assault, it could be claimed that what seemed to be an enactment of the assault as observed in the new seizures type was a déjà vu experience or false reminiscences as describe by Hughlings Jackson (Hughlings-Jackson, 1880). Such false reminiscences can even lead in rare cases to a PTSD-like clinical picture (Stewart and Bartucci, 1986; Cohen et al., 2010). Second, we limited our analysis to the fear circuitry of PTSD and did not assess other potential circuits involved in PTSD, such as emotional regulation and context processing (Sheynin and Liberzon, 2017). This was done since the fear circuitry is the foremost neural circuitry documented in PTSD. Third, in theory, we might have been able to test the causal interaction between the two disorders (PTSD and TLE) through an experimental activation of the patient seizures or PTSD. Nevertheless, due to obvious ethical considerations, this could not have been done. This is particularly misfortunate considering that hyper amygdala activation is a predisposing risk factor for PTSD (Admon et al., 2009, 2013a,b), and thus might have been present prior to the traumatic occurrence. Fourth, we were unable to assess the circuitry of the TLE as in an EEG-fMRI study and thus were not able to capture epileptic activity simultaneously with fMRI. Despite these limitations, our unique case of a patient exhibiting a change in seizure semiology following a traumatic event, and the extensive clinical and neural evidence that we were able to gather on her, enabled us to contribute critical evidence toward informed explanations of “pseudo” PNES.

## Conclusion

In this case study, we presented a patient with TLE accompanied by “pseudo” PNES (i.e., a PNES-like presentation with corresponding epileptic activity). We suggest that the anatomical proximity of the PTSD and the TLE brain circuitry enabled the simultaneous co-activation of both disorders. This unique presentation can shed light on the mechanisms involved in PNES. Further studies looking at this complicated and bidirectional interaction of TLE with anxiety in general and with PTSD in particular are warranted.

## Data Availability Statement

All datasets generated for this study are included in the article/supplementary material.

## Ethics Statement

The studies involving human participant were reviewed and approved by The Helsinki committee at Tel Aviv Sourasky Medical Center. The patient/participant provided her written informed consent to participate in this study. Written informed consent was obtained from the individual(s) for the publication of any potentially identifiable images or data included in this article.

## Author Contributions

IB, RA, TG, and FF wrote the manuscript. TG and RA analysed the functional imaging data. FF analysed the EEG and video monitoring data. IB and FF evaluated the patient.

## Conflict of Interest

The authors declare that the research was conducted in the absence of any commercial or financial relationships that could be construed as a potential conflict of interest.
